# The Macrophages-Microbiota Interplay in Colorectal Cancer (CRC)-Related Inflammation: Prognostic and Therapeutic Significance

**DOI:** 10.3390/ijms21186866

**Published:** 2020-09-18

**Authors:** Silvia Mola, Chiara Pandolfo, Antonio Sica, Chiara Porta

**Affiliations:** 1 Department of Pharmaceutical Sciences, Università del Piemonte Orientale “Amedeo Avogadro”, 28100 Novara, Italy; silvia.mola@uniupo.it (S.M.); chiara.pandolfo@uniupo.it (C.P.); chiara.porta@uniupo.it (C.P.); 2 Center for Translational Research on Autoimmune & Allergic Diseases (CAAD), Università del Piemonte Orientale “Amedeo Avogadro”, 28100 Novara, Italy; 3 Humanitas Clinical and Research Center (IRCCS), 20089 Rozzano (MI), Italy

**Keywords:** tumor-associated macrophages (TAMs), colorectal cancer (CRC), colitis-associated cancer (CAC), microbiota, cancer immunotherapy, tumor microenvironment (TME), prognostic biomarkers, predictive biomarkers

## Abstract

Tumor-associated macrophages (TAMs) are the main population of myeloid cells infiltrating solid tumors and the pivotal orchestrators of cancer-promoting inflammation. However, due to their exceptional plasticity, macrophages can be also key effector cells and powerful activators of adaptive anti-tumor immunity. This functional heterogeneity is emerging in human tumors, colorectal cancer (CRC) in particular, where the dynamic co-existence of different macrophage subtypes influences tumor development, outcome, and response to therapies. Intestinal macrophages are in close interaction with enteric microbiota, which contributes to carcinogenesis and affects treatment outcomes. This interplay may be particularly relevant in CRC, one of the most prevalent and lethal cancer types in the world. Therefore, both macrophages and intestinal microbiota are considered promising prognostic indicators and valuable targets for new therapeutic approaches. Here, we discuss the current understanding of the molecular circuits underlying the interplay between macrophages and microbiota in CRC development, progression, and response to both conventional therapies and immunotherapies.

## 1. Introduction

Beyond cancer cells, the composition of the tumor microenvironment (TME) is widely recognized as the driving force of solid tumors, influencing their development, growth, progression, and response to therapy. Within the TME, infiltrating immune cells are important actors that can either exert beneficial or detrimental activities [1,2]. Indeed, while immune cells can potentially recognize and eliminate tumor cells, they can also generate a “smoldering” inflammation, instrumental to tumor growth and progression [3,4]. Accordingly, the characterization of “immune landscape”, namely type, density, and location of immune cells within a tumor, is increasingly being recognized for its prognostic and predictive value. Notably, colorectal cancer (CRC) was the first tumor type for which the prognostic value of a T cells-based Immunoscore has been pointed out as a superior prognostic indicator, compared to the traditional TNM system [5]. The activity of T cells is tightly regulated by myeloid cells, which are also important sculptors of non-immune components of TME, such as blood vessels, stromal cells, and extracellular matrix [1]. Tumor-associated macrophages (TAMs) are the most abundant population of myeloid cells infiltrating solid tumors. Despite macrophages can be both powerful effectors and crucial initiators of the immune response, inside the tumors, they generally acquire an immunosuppressive, pro-angiogenic, and pro-metastatic phenotype, therefore acting as crucial tumor promoters [6,7]. Macrophages are widely recognized for their exceptional plasticity, namely the ability to change their functional phenotype in response to the dynamic changes of microenvironmental signals [8]. The M1-M2 dichotomy represents a clear and useful simplification of macrophage ductility, in vitro. Bacterial product (LPS) and Th1 cytokine (IFNγ) induce pro-inflammatory, cytotoxic, and antigen-presenting activities (M1-polarized activation), whereas the Th2 (IL-4 ± IL-13) cytokines promote the expression of an alternative (M2) program of polarized activation that supports immunomodulatory, pro-resolving, and pro-angiogenic functions [9]. Under both physiological and pathological conditions, the coexistence of multiple signals obviously results in a much more complex phenotype [10]; therefore, in vivo, macrophage plasticity emerges as a continuum of heterogeneous functional states, variably endowed of pro-inflammatory and effector functions (M1-skewed) or immunosuppressive and pro-healing properties (M2-skewed) [11,12].

An additional level of complexity arises from the distinct origins of the different subsets of macrophages. Over the last decade, fate-mapping studies in mice have demonstrated that many tissue-resident macrophages (TRMs) are a self-maintaining population of embryonic origin, which variably coexists with macrophages that are derived from adult circulating monocytes [13,14]. Albeit macrophage ontogeny has been mainly studied in mice, accumulating evidence based on transcriptomic profiling by single-cell RNA sequencing has confirmed the existence of various types of embryonic TRMs in human tissues (e.g., head, liver, lung, and skin) and has started to dissect the spatiotemporal dynamics of early macrophage development during human embryogenesis [15]. Although gut macrophages are thought to be exclusively replaced by circulating monocytes, recent studies have highlighted TRM populations of embryonic origin. Flow cytometric phenotyping and fate-mapping studies in mice have indicated the existence of a population of early seeded tissue-resident intestinal macrophages that display little to no turnover as the animals’ age [16,17]. Accordingly, a population of long-lived macrophages has been observed in human patients receiving intestinal transplants [18]. Therefore, in the intestine of both mice and humans, long-lived TRMs of embryonic origin coexist with macrophages readily replaced by circulating monocytes, through a process known as the ‘monocyte waterfall’ [19,20].

After seeding tissues, the local microenvironmental cues sculpt the transcriptional landscape of TRMs, which, in turn, express important trophic functions, supporting the development and homeostatic activity of the tissues in which they are located [21]. The epigenetic signature also constitutes a sort of “memory”, which modulates the response of TRMs to the new environmental cues. Consequently, in inflamed tissues, bone marrow-derived macrophages are functionally distinct from TRMs and impact differentially the outcome of multiple disorders [22,23]. This macrophage heterogeneity is increasingly appreciated also in cancer. Although in many tumor types, the majority of TAMs results from circulating monocytes, recent evidence indicates that in brain, lung, and pancreatic duct cancers, a significant percentage originates from TRMs [24,25,26,27]. To what extent the developmental origin characterizes the functional heterogeneity of TAM populations is an outstanding issue that the advent of single-cell based approaches will likely help to figure out. So far, strategies based on depletion, M1 repolarization, or promotion of the phagocytic activity of TAMs have been proven to be therapeutically effective in numerous pre-clinical studies that pave the way for their clinical development [28,29]. Although such macrophage-targeted approaches might be beneficial per se, its combination with other therapeutics will likely improve anti-cancer efficacy. Beyond contributing to tumorigenesis, TAMs can profoundly affect the response to anti-cancer therapies, including chemotherapy, radiotherapy, angiogenic inhibitors, as well as immunotherapies [30,31,32,33]. Undoubtedly, the unleashing of pre-existing anti-tumor immune responses through the blockage of the immune checkpoints—cytotoxic T-lymphocyte-associated protein 4 (CTLA-4) and programmed cell death protein 1 (PD-1)/PD-ligand 1 (PD-L1)—represents the breakthrough of cancer therapy of the last decade [34]. After the approval of ipilimumab (targeting CTLA-4) and pembrolizumab and nivolumab (targeting PD-1) for the treatment of metastatic melanoma, the clinical benefit of immune checkpoint blockers (ICBs) have been quickly appreciated for a growing number of cancer types, including non-small cell lung cancer, microsatellite instability-high (MSI-H) CRC, gastric cancer, Hodgkin lymphoma, head and neck squamous cell, hepatocellular, renal cancer, Merkel cell, and urothelial carcinoma [35]. Despite these broad successes in several malignancies, ICBs are now only useful on a small fraction of cancer patients, so the challenge at the forefront is to identify new predictors of response and understand how to overcome resistance mechanisms to enable a personalized approach of precision medicine. So far, many patient intrinsic characteristics (e.g., age, sex, HLA genotype, and genetic polymorphisms) and tumor intrinsic determinants (such as tumor mutational burden and TME composition) have been found associated with ICB sensitivity [36,37,38]. ICBs could be effective, providing that tumor cells express immunogenic antigens recognized by the immune system. Hence, the extent of tumor mutational burden (TMB) correlates with the probability of expressing newly formed antigens and ICB efficacy, as well as with higher benefits to ICB treatments [39,40]. The presence of an abundant T cell infiltrate and the expression of an IFNγ-signature, along with the expression of PD-1 and PD-L1, are additional favorable predictors of response to ICBs since they represent indicators of a pre-existing anti-tumor immunity [38]. Conversely, due to their immunosuppressive activities, myeloid cells are recognized as major brakes for ICB efficacy [41,42,43]. Accordingly, the combination of macrophage-targeted approaches with ICBs shows promising results in different pre-clinical models [44,45]. The therapeutic efficacy of ICBs can be also influenced by different environmental factors, such as the diet and the microbiome. A large body of evidence has shown that the gut microbiome makes substantial contributions to some types of cancer, in particular gastrointestinal cancers, through both direct effects and bystander immunomodulatory activities [46].

Here, we examine the role of macrophages in CRC development, focusing on the main molecular determinants of their pro-tumor activity, including the gut microbiota, as well as on their prognostic and predictive significance of the response to therapy, including ICBs.

## 2. Molecular Pathways Underpinning the Pro-Tumor Activity of Macrophages in CRC

CRC is the third most common cancer type and a leading cause of cancer-related deaths [47]. Although early diagnosis is usually associated with a favorable outcome, CRC is often detected at more advanced and, therefore, more challenging stages for treatment [48,49].

CRC frequently arises in patients with inflammatory bowel disease (IBD), including both Crohn’s disease and ulcerative colitis [50,51,52], and progresses with the help of macrophages that sequentially participate in IBD and CRC development and in the subsequent formation of an inflammatory TME. Accordingly, genetic inactivation of the signal transducer and activator of transcription 3 (Stat3) in macrophages, leading to the inactivation of anti-inflammatory IL-10 signaling, has resulted in chronic intestinal inflammation and onset of tumor lesions [53].

Although a strong causal association exists between IBD and the development of colitis-associated cancer (CAC), this causality accounts for only 2% of all CRCs [54], while in many cancers, oncogenic mutations occur in the absence of pre-existing inflammation. A small percentage of CRCs (about 5%) can be etiologically associated with hereditary cancer syndromes, among which the most common is familial adenomatous polyposis and hereditary nonpolyposis colorectal [55,56]. An additional 10-30% of CRCs have a family history, indicating the existence of an inherited genetic susceptibility, while up to 75% arise sporadically through sequentially acquired genetic and epigenetic aberrations [57].

Despite the majority of CRCs arises in the absence of an obvious inflammatory process, neoplastic transformation is associated with the construction of an inflammatory tumor-promoting microenvironment [4,50]. Indeed, the discovery of tumor-suppressive effects of nonsteroidal anti-inflammatory drugs (NSAIDs) dates back to 1981 [58], and aspirin hitherto remains the most effective chemopreventive drug [49]. Albeit the adverse cardiovascular effects limit its widespread prophylactic use, selective targeting the PGE2/EP signaling, downstream to the enzymatic activity of cyclooxygenases (COX), represents a potential alternative approach with less severe adverse effects and has recently entered in the clinical trial (NCT02540291) [49].

Independent on CRC origin, TAMs are crucial regulators of tumor-promoting inflammation. Indeed, the blockade of the CCL2/CCR2 axis, in both a preclinical model of CAC and a genetic model of intestinal tumorigenesis (Apc^Min^ mice), leads to the reduction of TAMs in association with significant inhibition of tumor multiplicity and growth [59,60].

**Figure 1:**
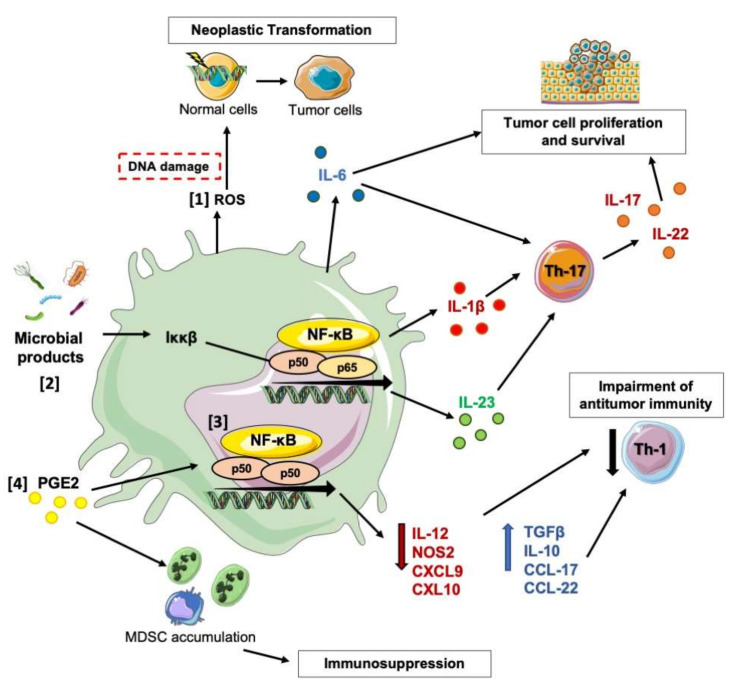
Mechanisms and Functions Underlying the Pro-tumor Activities of Macrophages in CRC. TAMs are key orchestrators of tumor-promoting inflammation. [1] Macrophages-derived ROS induces DNA damage and mutations in neighboring epithelial cells, supporting neoplastic transformation and malignant progression. [2] Commensal bacteria and microbial products activate the expression of inflammatory cytokines via NF-κB. IL-6, IL-1β, IL-23 promote the proliferation and survival of neoplastic cells, as well as differentiation of pro-tumorigenic Th-17 T cells. [3] During tumor development, nuclear p50 NF-κB accumulation in TAM drives a shift in the polarized inflammatory response from type 1 (IL-12, iNOS, CXCL-9, CXCL-10) to type 2 (TGβ, IL-10, CCL17, CCL22). This event creates tumor-promoting conditions by hampering the cytotoxic actions of the Th1/M1-polarized immune response. [4] Prostaglandin E2 (PGE2) exerts a pivotal role in the generation of an immunosuppressive TME by promoting expansion and activation of myeloid-derived suppressor cells (MDSC) and M2-skewed macrophage polarization.

Several studies have shed light on the molecular mechanisms whereby macrophages drive neoplastic transformation, growth, and spread (Figure 1). Albeit intestinal epithelial cells can autonomously produce reactive oxygen species (ROS) and reactive nitrogen species (RNS), the idea that inflammatory cells could induce DNA damage and mutations through the production of ROS and RNS has long been suggested [61]. Accordingly, recent in vivo studies have proven that myeloid-derived ROS can promote both initial neoplastic transformation of epithelial cells and their subsequent malignant progression. Indeed, in mice with increased ROS production by myeloid cells, chronic dextran sulfate sodium (DSS)-induced inflammation is sufficient to trigger the onset of CRC lesions, and treatment with the carcinogen azoxymethane leads to the development of invasive tumors, rather than benign adenomas [62]. Beyond oxidative stress, myeloid cells are a crucial source of inflammatory cytokines, which can support the survival and proliferation of neoplastic cells (e.g., IL-6, IL-1, IL-23, IL-17A) or the activation of adaptive anti-tumor immunity (e.g., IL-12, IFNγ). The transcription factor NF-κB is a master regulator of the expression of inflammatory cytokines, anti-apoptotic, and pro-cycling genes, therefore representing a crucial connector between inflammation and tumorigenesis [63]. Accordingly, blockage of IKKβ-dependent NF-κB activation in intestinal epithelial cells impairs proliferation, survival, and epithelial-mesenchymal transition (EMT) of neoplastic cells, leading to important anti-tumor effects in both models of CAC and carcinogen-induced CRC [64,65]. The impact of myeloid-specific NF-κB activation on CRC development is more complex since the formation of different NF-κB dimers induces distinct macrophage activation states [66], which are associated with either tumor promotion or resistance [67,68]. Exemplary, while in a CAC model, IKKβ-dependent activation of NF-κB in myeloid cells promotes tumor growth through the production of IL-6, which stimulates cancer cell proliferation and survival via STAT3 [69,70], we have observed that nuclear accumulation of p50 NF-κB in TAMs supports tumor-promoting inflammation. In particular, while CRC lesions have shown increased expression of M2-related (Il10, Tgfb1, Ccl17, and Ccl22) and tumor-promoting genes (Tnf and Il23a), genetic ablation of p50 has impaired IL-23 expression and enhanced M1/Th1 immune responses, leading to a significant reduction of tumor multiplicity and growth in models of both CAC and genetically-induced intestinal tumorigenesis [71]. Interestingly, the importance of M1-polarized myeloid cells to restrain CRC development emerges also in the absence of IKKα and mechanistically requires the IKKβ-driven activation of NF-κB in intestinal epithelial cells, along with the subsequent production of monocyte-recruiting chemokines [72].

Beyond the direct pro-tumor activity on cancer cells, PGE2 signaling in infiltrating immune and stromal cells enhances the generation of an immunosuppressive TME [73]. Interestingly, PGE2 triggers both p50 NF-κB accumulation in macrophages [67] and their M2-skewed polarized activation. Similar to p50^−/−^ mice, myeloid-specific deletion of PGE2 receptor EP4 or its pharmacological inhibition has led to a significant reduction of genetically-induced intestinal tumorigenesis (Apc^Min^ mice), in association with increased frequency of M1-anti-tumor macrophages [74]. PGE2 is crucial also for the expansion and activation of myeloid-derived suppressor cells (MDSCs) [75,76]. Inhibition of PGE2/EP2 signaling by either aspirin or the PGE2 receptor EP2 antagonist AH6809 impairs both accumulation and immunosuppressive activity of CD11b^+^Gr1^+^ MDSCs, leading to a significant reduction of CAC development [76]. Notably, we have recently demonstrated that tumor-derived PGE2 drives the nuclear accumulation of p50 NF-κB in CD11b^+^Ly6G^-^Ly6C^high^ M-MDSC, diverting their response to IFNγ towards NO-mediated immunosuppression and reducing TNFα expression [77]. Consistently, the ablation of p50, as well as pharmacological inhibition of EP2 by AH6809, has reprogrammed M-MDSC towards a NOS2^low^/TNFα^high^ phenotype, restoring the in vivo anti-tumor activity of IFNγ [77]. Further, circulating M-MDSCs (CD33^+^CD14^+^HLA-DR^low/−^ cells) of CRC patients express the EP2 receptor and show increased nuclear accumulation of p50 in association with the elevated expression of the immunosuppressive NOS2 [77].

Along with the suppression of anti-tumor immunity, myeloid cells produce inflammatory cytokines (IL-23, IL-6, IL-1) that drive the expansion and activation of pro-tumorigenic T helper 17 (Th17) cells [78,79]. In a mouse model of colorectal tumorigenesis, the barrier defects induced by initiating lesions lead to the translocation of commensal bacteria and microbial products that trigger myeloid cells to release a copious amount of IL-23 [80]. In turn, the immunomodulatory properties of IL-23 have favored CRC development by inducing a tumoral IL-17 response, associated with the inhibition of NK cell effector functions [81] and enhanced expansion and activation of Th17 cells. Several studies indicate that high levels of IL-17 enhance colorectal tumorigenesis [82] by activating cancer cell proliferation and survival [80,83]. However, despite in early-stage human CRC, elevated expression of IL-23 [84] and IL-17A [85] is recognized as unfavorable prognostic indicators, elevated expression of IL-17A in advanced CRC appears to be associated with improved outcome [86], suggesting that, at some point, IL-17A may stimulate anti-tumor immunity. Similarly, a dual activity of IL-1 has been reported in CRC. In spite of a pro-tumorigenic activity of IL-1 is recognized in different human cancer types [87], IL-1 signaling in distinct CRC-infiltrating immune cells is associated with the expression of both pro- and anti-tumorigenic activities [88]. In the preclinical model of CRC, specific inactivation of IL-1R in cell types other than myeloid cells has confirmed that IL-1 signaling promotes proliferation and survival of neoplastic cells and drives both Th17 differentiation and production of pro-tumorigenic cytokines (IL-17 and IL-22) [88]. In contrast, IL-1 signaling in myeloid cells is anti-tumorigenic. Indeed, lack of Il1r1 in neutrophils promotes tumor-associated dysbiosis, with a consequently heightened infiltration of bacteria into the tumor tissue, increased production of pro-tumorigenic cytokines by TAMs, and aggressive CRC progression [88].

## 3. Cross-Talk between Gut Microbiota and Macrophages in CRC Development 

Beyond the cumulative pro-tumorigenic effects associated with the augmented bacterial translocation into neoplastic tissue [80], CRC development is causally associated with gut dysbiosis [46]. Characteristic alterations of the fecal microbiota are, in fact, detected in CRC patients compared to healthy subjects, as well as in the tumor (both adenoma and cancer) compared to the adjacent healthy mucosa [89,90,91]. Moreover, the transplant of stool samples from CRC patients or healthy subjects in germ-free or microbiota-depleted mice has demonstrated that fecal microbiota of CRC patients selectively increases chemical-induced polyps [92]. Mechanistically, fecal microbiota transplantation (FMT) of CRC bearers support tumor development by increasing the expression of pro-tumorigenic cytokines (e.g., IL-17A, IL-22, and IL-23), inflammatory chemokines (e.g., CCL-1, CCL-2, CCL-3, CCL-4, CXCL-12), and genes involved in the regulation of cell cycle, stemness, apoptosis, angiogenesis, tumor invasiveness, and metastasis [92]. In addition to bacterial dysbiosis, characteristic alterations of both fecal virome [93,94] and mycobiome [95] have been observed in CRC patients, suggesting that along with bacteria, virus and fungi might be exploited as novel non-invasive markers for early CRC detection. Therefore, the understanding of the pathogenetic mechanisms underlying the pro-tumorigenic activities of the various microbial components could highlight new targets for therapeutic interventions.

**Figure 2:**
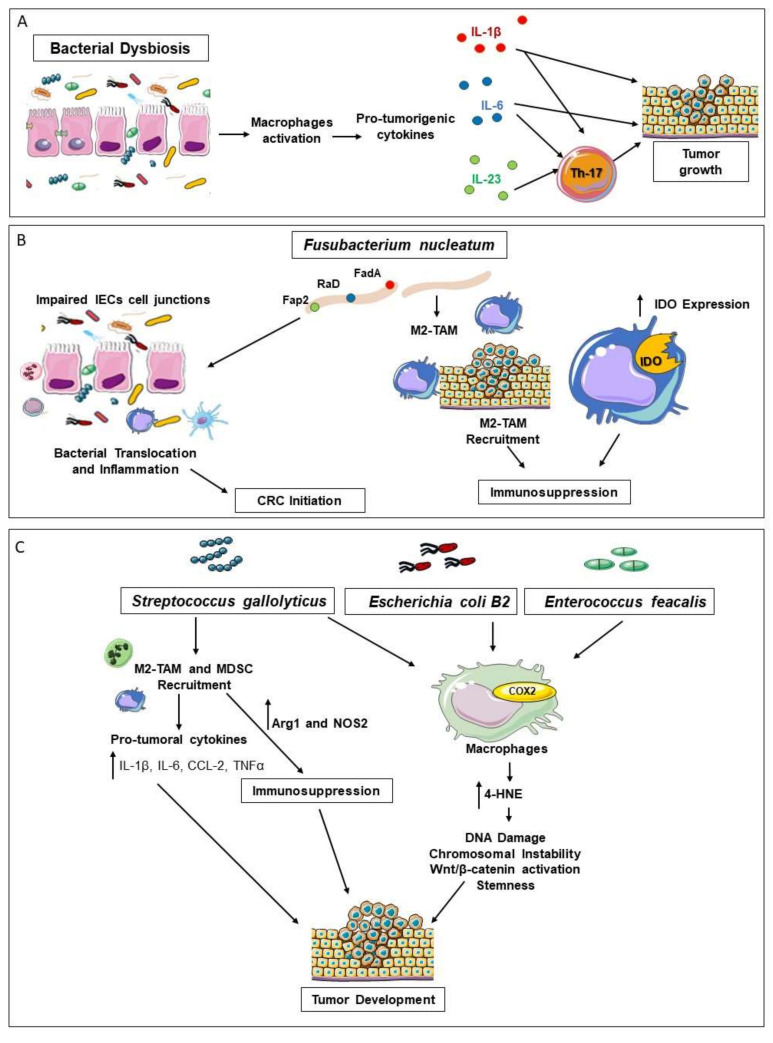
The Interplay of Macrophages and Microbiota in CRC Development. Intestinal microbiota can sustain carcinogenesis through macrophage-induced bystander effects. (**A**) The barrier defects associated with oncogenic transformation lead to the translocation of commensal bacteria and microbial products that trigger myeloid cells to release tumor-supporting inflammatory cytokines (e.g., IL-6, IL-1β, and IL-23). Additionally, these cytokines trigger the expansion and activation of pro-tumorigenic Th17 cells. (**B**) *Fusobacterium nucleatum* promotes the initial phase of CRC development through the production of virulence factors (e.g., FadA, Fap2, RaD) that impair colonic epithelial cell junctions, favoring its translocation and the instauration of an inflammatory tumor-promoting microenvironment. During CRC development, *F. nucleatum* supports the generation of an immunosuppressive TME by favoring the selective intra-tumor recruitment of M2-like TAMs and MDSCs. Macrophages infected by *F. nucleatum* in vitro show an increased surface expression of the immunoregulatory enzyme indoleamine 2,3-dioxygenase (IDO). (**C**) *Streptococcus gallolyticus* is enriched in colonic carcinoma, promoting the selective accumulation of tumor-infiltrating myeloid cells. In turn, TAMs and MDSCs inhibit T cells via Arg1 and iNOS, hence supporting the generation of an immune-suppressive microenvironment favorable to tumor progression. *S. gallolyticus, Enterococcus faecalis,* and B2 phylogenetic group of *Escherichia coli* infect macrophages and stimulate COX-2 expression. In addition to PGE2, COX2 generates 4-hydroxy-2-nonenal (4-HNE), a diffusible breakdown product of ω-6 polyunsaturated fatty acids, which causes DNA damage, chromosomal instability, dedifferentiation, and reprogramming of primary colon epithelial cells.

So far, most studies have focused on the most relevant bacterial components of the microbiota (Figure 2).

Intestinal bacteria can contribute to CRC development, progression, and response to therapy either directly, via metabolic activation of carcinogens and mutagen products (e.g., environmental polyamine, phenols, and alkylating agents), or indirectly through the modulation of immune cell functions [96,97]. Overall, bacterial dysbiosis weakens the intestinal barrier, favoring bacterial translocation, macrophages activation, and the consequent establishment of chronic pro-tumorigenic inflammation [98]. Indeed, the use of antibiotics in both CAC and genetic models of intestinal tumorigenesis drastically reduces intestinal inflammation and CRC development [99,100].

Multiple lines of evidence indicate the existence of a tight relationship between enteric bacteriome, macrophages, and tumor promotion [96]. Macrophages of mice with intestinal dysbacteriosis release pro-tumorigenic cytokines (e.g., IL-6 and TNF) and stimulate in vivo the growth of tumor xenografts, while promoting colon cancer cell proliferation and EMT in vitro [101]. Notably, depletion of macrophages totally abrogates the pro-tumor effect of intestinal dysbacteriosis, indicating that bacteria require macrophages to exert pro-tumor activities [101,102]. Moreover, macrophages can drive alterations of the microbial profile associated with CRC promotion. Indeed, in a CAC model, Bader and colleagues demonstrated that late macrophage depletion inhibits onset and growth of tumor lesions, in association with reduced expression of pro-tumorigenic cytokines (e.g., IL-6, IL-13, IL-10, TGFβ, and CCL-17) and expansion of Firmicutes, a phylum endowed of anti-tumorigenic effects [103]. The release of microbial outer membrane vesicles (OMVs) is a relevant mechanism whereby bacteria sustain carcinogenesis, through both direct and macrophage-induced bystander effects [104]. On the one hand, OMVs transfer genetic material to tumor cells and, on the other, the OMV engagement of TLR2/TLR4 on epithelial cells triggers the release of exosomes, which, in turn, induces macrophages to produce pro-tumor cytokines (e.g., IL-6 and IL-18) [105].

Enteric microbiota of CRC bearers is characterized by an overall reduction of bacterial diversity, leading to the enrichment of selected bacterial species that engage macrophage-driven pro-tumorigenic activities. For example, *Fusobacterium nucleatum* promotes the initial phase of CRC development by producing virulence factors (e.g., FadA, Fap2, RadD) that impair colonic epithelial cell junctions and favor its translocation. This event triggers the recruitment and activation of inflammatory cells, building up an inflammatory microenvironment that fosters neoplastic transformation [106]. During tumor development, *F. nucleatum* supports the selective recruitment of M2-like TAMs and MDSCs, leading to the generation of an immunosuppressive TME favorable to tumor growth and progression [107]. Mechanistically, *F. nucleatum* triggers TAM activation through the engagement of TLR4 and the subsequent activation of IL-6/STAT3/c-MYC signaling, supporting their M2-skewed polarization [108]. Macrophages infected by *F. nucleatum* upregulate IDO on the cell surface, suggesting an additional mechanism whereby *F. nucleatum* might trigger macrophage-driven immunosuppression [106].

*Prevotella* and *Porphyromonadacea* are bacterial genera, which undergo expansion in a murine model of CAC and enhance the release of pro-tumorigenic cytokines (e.g., TNF-α, IL-6, IL-1β, and IL-23) by immune cells [18]. In line, the enrichment of *Prevotella* and *Porphyromonadacea* in tumor bearers correlates with the extent of pro-tumorigenic activity that can be transferred via FMT in germ-free mice [99].

*Enterococcus faecalis*, *Streptococcus gallolyticu*s, and B2 phylogenetic group of *Escherichia coli* exert their pro-tumorigenic activity via macrophage-induced bystander effects. They infect macrophages and stimulate the expression of COX-2 and PGE2 expression, which, in turn, favors tumor onset and growth [109,110,111]. *E. faecalis*-infected macrophages release clastogens that, in addition to causing DNA damage and chromosomal instability [112], favor cancer cell stemness [113,114]. For example, 4-hydroxy-2-nonenal (4-HNE) is a DNA mutagen and mitotic spindle inhibitor that is generated from ω-6 polyunsaturated fatty acids via COX2 [102]. 4-HNE also induces the activation of the Wnt/β-catenin pathway and the expression of multiple pluripotent transcription factors (e.g., c-Myc, Klf4, Oct4, and Sox2) in murine primary colon epithelial cells (YAMC). Similarly, exposure of YAMC to *E. faecalis*-infected macrophages enhances Wnt/β-catenin activation and expression of cancer stem cell markers (e.g., CD44, DCLK1), indicating that the interplay between *E. faecalis* and macrophages is directly involved in dedifferentiation, reprogramming, and malignant transformation of primary colon epithelial cells [114].

In a CAC model, oral pre-treatment with *S. gallolyticus* exacerbates both inflammation (e.g., IL-6, IL-1β, IL-8, CCL2, TNFα) and tumor formation. As compared to healthy colon and adenoma, *S. gallolyticus* is enriched in colonic carcinoma, where it promotes the selective recruitment of TAMs and MDSCs, which inhibit T cells via Arg1 and NOS2 and support the generation of an immune-suppressive microenvironment [115].

Although most studies aimed at identifying pro-tumorigenic bacterial species have focused on strains that enrich themselves in cancer carriers (e.g., *F. nucleatum, S. gallolyticus, Bacteroides fragilis, E. coli, E. faecalis*) [116,117,118], CRC dysbiosis is also associated with the decrease of beneficial species (e.g., *Bifidobacteria* and *Lactobacilli*), suggesting that selective loss of anti-tumorigenic bacteria might be an additional mechanism contributing to tumor development. According to this hypothesis, Zagato and colleagues have recently identified two strains of bacteria—*Faecalibaculum rodentium* in mice and its human homolog *Holdemanella biformis*—which are under-represented in tumor bearers and can actually counteract CRC development and progression [119]. Mechanistically, both bacteria produce short-chain fatty acids that inhibit histone H3 deacetylation, hampering calcineurin and NFATc3 activation and tumor cell proliferation [119].

## 4. The Interplay between Dietary Habits, Intestinal Microbiota, and Macrophages in CRC

Gut microbiome composition is strictly influenced by different external factors, including diet [120], physical activity [121], and alcohol consumption [122].

Dietary fibers promote the expansion of beneficial bacteria species, such as *Lactobacilli* and *Bifidobacteria* [123], which metabolize non-digestible carbohydrates in SCFAs (e.g., propionate, acetate, butyrate) [124]. In turn, SCFAs exert crucial immunomodulatory and anti-carcinogenic activities [109,123]. Butyrate can modulate the immune response of colonic macrophages through the inhibition of histone deacetylases, with a potential contribution to the maintenance of immunological tolerance to commensal microorganisms [125]. Exposure of mouse macrophages to butyrate downregulates LPS-induced pro-inflammatory mediators (e.g., IL-6, IL-12, and NO), restoring intestinal immune homeostasis [125,126]. Accordingly, butyrate administration, in vivo, can mitigate intestinal inflammation and lesions in both IBD patients and murine models [127,128]. Moreover, the combination of SCFAs with diet- and gut microbiota-derived indole derivatives modulates the susceptibility to intestinal inflammation in macrophages [129]. Some fiber-containing food is also rich in bioactive plant-derived phytochemicals, such as quercetin that is endowed of anti-inflammatory and anti-carcinogenic effects [128]. In line, quercetin administration in ApcMin mice has lowered polyposis in association with a reduced macrophage infiltration [130]. Alternate day fasting, in mice, inhibits colon carcinoma cell growth, without causing a reduction of body weight, but suppressing M2 TAM polarization through the decreased generation of extracellular adenosine and consequent inactivation of JAK1/STAT3 signaling pathway [131]. These studies strengthen the concept that dietary components and interventions exert crucial effects in CRC by shaping macrophage activity. In contrast to the beneficial activity of fiber, a diet enriched in saturated fats, refined carbohydrates, and red and processed meat, own pro-inflammatory properties and, together with obesity and low physical activity, are recognized as key exogenous factors in CRC etiology [132].

Both the preclinical model of CRC (*Apc^Min^ mice*) and clinical studies have demonstrated that a high-fat diet (HFD) increases the incidence of CRC. Mechanistically, HFD induces dysbiosis that supports adenoma-adenocarcinoma sequence through the CCL2/CCR2-dependent accumulation of pro-tumoral, M2-polarized TAMs [133]. High intake of red and processed meats is also associated with a high intake of preservatives (such as nitrates and nitrites) and carcinogenic chemicals produced during meat processing and cooking, such as heterocyclic amines and polycyclic aromatic hydrocarbons [134]. Additionally, nutrients enriched in red meat, such as choline and carnitine, are metabolized by gut microbiota in products (e.g., trimethylamine and trimethylamine N-Oxide) that have been associated with an increased risk of CRC [135,136].

Overnutrition and imbalanced diets contribute to obesity, a chronic inflammatory status associated with a significant decrease in the diversity of the gut microbiota, including a significant reduction of beneficial *Bacteroides* species [137]. In addition, diet-induced obesity enhances chemically-induced CAC in mice by heightening inflammation. In particular, high IL-6 production skews macrophage activation towards a tumor-promoting phenotype, which, in turn, favors CCL20-dependent immune cell recruitment and CAC development [138].

The importance of a healthy lifestyle for CRC prevention is strengthened by the observation that individuals with the highest level of physical activity have a lower risk of developing CRC [121]. Conversely, wrong lifestyle behaviors, such as alcohol intake, is recognized as a major risk factor for CRC development. Beyond the carcinogenic effects of its metabolites, ethanol can directly induce intestinal inflammation through multiple pathways [139]. Ethanol increases intestinal permeability, as well as microbial dysbiosis, bacterial overgrowth, and alterations in the mucosal immune system [139,140]. Gut barrier dysfunction results in increased exposure of immune cells to LPS, leading to a pro-tumorigenic inflammatory response, exacerbated production of ROS, and cytokines, such as IL-6 and IL-18 [139,141]. In addition, chronic ethanol feeding increases AOM/DSS-induced CAC by enhancing immune cell infiltration and inflammatory cytokines production [142].

Altogether, this evidence demonstrates an important impact of diet on microbiota composition and activation status of macrophages, suggesting its crucial role in the pathogenesis of intestinal inflammation and in the development of CRC [143].

## 5. Macrophages as Prognostic and Predictive Biomarker in Human CRC

Although the pro-tumoral activity of TAM during CRC development has been clearly demonstrated by preclinical studies, their impact on human CRC progression is still controversial [144,145,146,147]. In contrast to most solid cancers [32], some studies indicate total TAM infiltration is found unable to predict outcome [148,149], suggesting that different states of activation of macrophage subsets could be decisive in human CRC. In line, simultaneous accumulation of M1- (NOS2^+^) and M2-polarized (CD163^+^) macrophage populations is observed in human CRC tumors [150,151]. A recent meta-analysis indicates that pan-macrophages (CD68^+^) are favorably associated with overall survival [152]; however, CD68 can occasionally be expressed in stromal and cancer cells themselves; therefore, the data obtained by using this marker should be carefully assessed. Moreover, patients’ stratification by macrophage subtypes highlights that a high density of CD163^+^ (M2-skewed) macrophages is associated with a poorer outcome [153]. In agreement, in two independent cohorts of consecutive CRC patients with pathologic stage II, a high frequency of M2-skewed macrophages, identified as a high CD206/CD68 ratio, is significantly associated with disease recurrence and shorter overall survival [154]. Further, the type of TAM infiltrate changes between MSI-H and MSI-low (MSI-L) tumors [155], suggesting that stratification by MMR status should be advisable to improve the prognostic value of TAM infiltrate [152].

An additional level of complexity is related to the spatial localization of TAM, as several pieces of evidence suggest that TAM may exert different functions in relation to their inner or peripheral tumor localization. In particular, TAMs located at the invasive front would exert beneficial activities [147,156], whereas intra-tumoral macrophages appear to play tumor-promoting roles [157] or be unable to predict outcome [152].

Combining the TAM location and functional phenotype could represent a strategy to improve their prognostic value. According to this, in a series of 150 CRC cases, the combination of CD68 as a macrophage lineage marker, CD80 as a marker of anti-tumor (M1) macrophages, and CD163 as a marker of pro-tumor (M2) macrophages has corroborated that distinct macrophage subtypes are differentially distributed throughout the tumor and are associated with different outcomes [158]. In line with previous studies, CD163 is observed to be expressed by almost 40% of TAMs, in particular those that are located at tumor invasive front [158]. In contrast to NOS2-expressing TAMs [151], the use of CD80 as a marker of M1-polarized macrophages has shown that, in comparison to adjacent healthy mucosa, the majority of intra-tumoral macrophages downregulate CD80 and are, therefore, skewed towards an immunosuppressive phenotype [158]. Indeed, within stage III tumors, higher CD68 infiltration in the intra-tumoral regions is associated with decreased overall survival, and a higher CD80/CD163 ratio at the tumor invasive front correlates with a favorable outcome [158]. 

Confirming the tumor-promoting activity of M2-skewed macrophages, recognizable by nuclear accumulation of p50 NF-κB, we have observed in a cohort of 49 CRC patients (stage II/III) that accumulation of p50^+^ TAMs at the invasive margin is negatively correlated with M1 (IL12A) and Th1 (TBX21) gene expression and is associated with worse outcome [71].

In vivo evaluation of macrophage plasticity and diversity is challenging. Whereas most of the current studies have used a single marker of polarized activation, new approaches that allow the simultaneous analysis of multiple markers (Table 1) could improve the prognostic significance of TAM subsets in CRC. For example, imaging mass cytometry using metal-tagged antibodies might be pursued for multiplex protein detection, enabling the identification of TAM subpopulations within the context of the tissue structure. Transcriptomic profiling by single-cell RNA-Seq (scRNA-Seq) analysis is a powerful approach, able to provide a comprehensive map of the different macrophage subtypes. Recently, the characterization of human and mouse CRC lesions by scRNA-Seq has identified distinct myeloid populations associated with a differential sensitivity to CSF1R blockade and responsiveness to anti-CD40 treatment [159]. TAM can also influence the effectiveness of cytoreductive therapies, either antagonizing or synergizing the anti-tumor activity of these treatments. Therefore, depending on the treatment, the prognostic value of TAM may change accordingly. For example, for stage II colon cancer, a high CD206/CD68 ratio is both an unfavorable prognostic biomarker and a positive predictive biomarker of response to postoperative adjuvant chemotherapy [154]. Similarly, within stage III tumors, higher CD68 infiltration of the invasive front is associated with a poor outcome [158], whereas, for patients that undergo to 5-fluorouracil (5-FU) adjuvant therapy, the extent of CD68+ TAM infiltration is positively correlated with the overall survival [154].

## 6. The Interplay of Macrophages and Microbiota in Conventional Anti-Cancer Therapies

Radiotherapy and chemotherapy remain the leading strategies for controlling tumor spread and growth in patients with advanced and inoperable CRC lesions. Therapy regimens based on the different combinations of 5-FU, oxaliplatin, irinotecan, and capecitabine are the backbone of CRC treatment.

Macrophages generally curtail chemotherapy efficacy by orchestrating a tumor-promoting response and by providing a protective niche for cancer stem cells. However, TAMs can also synergize with selected drugs, and, under certain conditions, an abundant TAM infiltrate enhances the chemotherapy efficacy [28]. This is the case of 5-FU, a chemotherapeutic capable of reprogramming macrophages towards an M1-anti-tumor phenotype [160]. Moreover, certain cytotoxic agents, such as oxaliplatin, can induce “immunogenic cell death” (ICD), which stimulates the uptake and presentation of tumor-associated antigen by DCs to T cells [161]. Although ICD can also be induced by radiotherapy, in irradiated mice, the monocytes recruited to the tumor generally differentiate into immunosuppressive and tissue repairing macrophages, thus contributing to tumor relapse [162,163]. In contrast, neoadjuvant low-dose irradiation limits this pro-tumoral differentiation of macrophages [164], suggesting that phenotypic maturation of macrophages is dependent on radiation-absorbed dose.

Multiple lines of evidence indicate that the outcome of anti-cancer therapies is also deeply influenced by gut microbiota [165]. Intestinal microbiota can both increase efficacy or toxicity of chemotherapy by different mechanisms, including modulation of drug metabolism, activation of inflammatory pathways, and host immune response [165,166]. For example, the *F. nucleatum* promotes autophagy in the CRC cells, supporting chemoresistance [167]. In contrast, *Clostridia* spp. produces glucoronidases that reactivate irinotecan in the distal intestine, contributing to the typical gastrointestinal side effects [168,169]. Gut bacteria are also essential for radiation-induced enteritis, and germ-free mice are resistant to the lethal gastrointestinal effects of radiation [170].

Microbiota exerts a shaping activity on tumor-associated myeloid cell functions [166]. In different tumor models (e.g., CRC, melanoma, lymphoma), the efficacy of platinum-based chemotherapy is strictly dependent on the presence of both gut microbiota and myeloid cells [171]. Platinum compounds are genotoxic drugs that act through the formation of DNA-adducts and the production of mitochondrial ROS by tumor-associated inflammatory cells and cancer cells themselves [171,172]. The activity of platinum compounds is enhanced by microbiota, which increases the paracrine production of ROS by inducing NADPH oxidase 2 (NOX2) in myeloid cells [171]. In line, either germ-free or microbiota-depleted tumor-bearing mice show an impaired response to platinum compounds, along with defective production of ROS by tumor-infiltrating myeloid cells [171]. Similarly, myeloid cell depletion hampers oxaliplatin efficacy, corroborating the existence of a symbiotic relationship between microbiota and the immune system [171].

## 7. The Interplay of Macrophages and Microbiota in ICBs-Based Immunotherapy

In the last decade, ICB-based immunotherapy has achieved unprecedented clinical results in many solid tumors [173,174,175]. Despite initial studies of ICB in CRC had given disappointing results [176,177,178], the stratification by TMB and immune infiltrate has highlighted the efficacy of PD-1 and CTLA4 neutralization in a small subgroup of CRC patients characterized by an MSI-H phenotype due to a deficient DNA mismatch repair (dMMR) system [179,180,181]. In these patients, the inactivation of one of the four MMR genes increases the mutational rate 20 times, leading in more than 80% of cases to a high TMB [155,182]. dMMR/MSI-H tumors are also largely infiltrated by immune cells, in particular CD8^+^ and T-helper 1 (Th1) CD4^+^ lymphocytes expressing high levels of CTLA-4 and PD-1, whereas myeloid cells expressing the immune checkpoint ligands (PD-L1) are mainly located at the tumor-stroma interface [183,184,185]. The gene signature of dMMR/MSI-H tumors includes type I interferons, pro-inflammatory cytokines, and Th1-recruiting chemokines (e.g., CXCL9 and CXC10), indicating that tumor intrinsic IFN-signaling is functional [186,187]. All together, these features indicate a pre-existing anti-tumor immunity that is hampered by the immune checkpoints and can be efficiently unleashed by ICBs. Accordingly, in 2017, the anti-PD-1 inhibitors pembrolizumab (KeytrudaVR, Merck) and nivolumab (OpdivoVR, Bristol-Myers Squibb) have been approved by the Food and Drug Administration (FDA) for the treatment of patients with dMMR/MSI-H CRC.

Unfortunately, dMMR/MSI-H CRC represents only a small fraction of all CRC, whereas the majority of patients harboring a proficient MMR (pMMR)/MSI-low (MSI-L) tumor do not benefit from ICB treatment alone [188]. Despite most of pMMR/MSI-L CRC has low TMB and is poorly infiltrated by either T cells or inhibitory ligand-expressing cells, 2–3% of pMMR/MSI-L tumors exhibit an ultramutated phenotype characterized by a high number of frameshift mutations [189]. This phenotype is due to the inactivation of DNA polymerase epsilon or delta (POLE, POLD), which are involved in DNA replication and repair [190,191]. The POLE-mutated pMMR/MSI-L tumors are also enriched in PD1^+^CD8^+^ T cells and PD-L1^+^CD68^+^ macrophages and express high levels of pro-inflammatory cytokines and immune checkpoints (PD-1, PD-L1, and CTLA-4) [192,193], overall suggesting that they can benefit ICBs [194]. In line, Jun and colleagues have recently reported the first case of clinical response to pembrolizumab from a treatment-refractory patient, harboring a POLE-mutated pMMR/MSI-L metastatic CRC [195,196].

**Figure 3:**
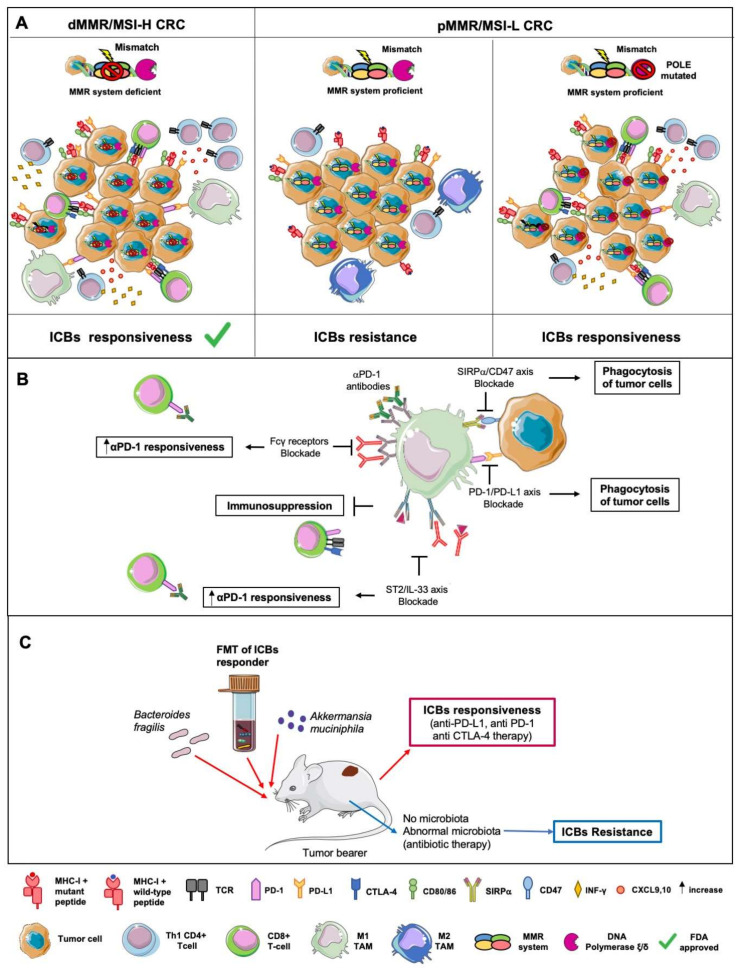
Responsiveness of CRC Patients to ICB-based Immunotherapy. Multiple determinants influence the efficacy of ICB-based immunotherapy in CRC patients (**A**). Overall, high TMB, high IFN signature, a massive infiltrate of PD-L1-expressing TAMs, CD8+ and Th1 CD4+ T-cells expressing high levels of CTLA-4 and PD-1 are recognized as crucial determinants of ICB responsiveness. Anti-PD-1 therapy is approved for dMMR/MSI-H tumors, whereas pMMR/MSI- L tumors are resistant. DNA polymerase epsilon or delta (POLE, POLD)-mutated CRC might be responsive to ICB due to an ultramutated phenotype associated with an elevated inflammatory cytokine gene expression and strong immune cell infiltration, enriched in CD8+ PD-1+ T cells and PD-L1+ TAMs. (**B**) TAMs control ICB responsiveness through multiple mechanisms. TAMs capture anti-PD-1 antibodies via Fcγ receptors, limiting their availability for tumor-infiltrating CD8+ T cells. Blockade of Fcγ receptors enables CD8+ T cells activation and cytotoxic tumor cell killing, increasing the therapeutic efficacy of ICBs. TAMs express different inhibitory receptors (e.g., PD-1 and Sirpα) that impair the uptake of tumor cells. Neutralization of PD-1/PD-L1 or Sirpα/CD47 axes restores macrophage-dependent tumor cell clearance and might increase ICB efficacy. The upregulation of ST2 by its ligand IL-33 supports TAM-suppressive activities. Pharmacological inhibition of the ST2/IL-33 axis augments the frequency and functions of intra-tumor CD8+ T cells and might act synergistically with anti–PD-1 therapy. (**C**) The composition of the gut microbiota affects the responsiveness to ICBs. FMT of ICB responsive patients or oral supplementation of selective bacteria species (*Akkermansia muciniphila* and *Bacteroides fragilis*) enhances the efficacy of PD-1 and CTLA-4 blockade in both germ-free and dysbiosis mice.

Beyond the molecular features of CRC, accumulating insights indicate TAMs control ICB responsiveness (Figure 3).

For example, using intravital imaging to follow fluorescently labeled PD-1 antibodies in CRC bearing mice, Arlauckas et al. have observed that TAMs capture anti-PD-1 via their Fcγ receptors, limiting its availability for tumor-infiltrating CD8^+^ T cells [42]. Consequently, the blockade of Fcγ receptors increases the therapeutic efficacy of tumor-infiltrating CD8^+^ T cells [42]. Beyond T cells, neutralization of PD-1/PD-L1 can also act through a direct effect on macrophages. Indeed, in both CRC mouse models and human patients, PD-1 has been found expressed by a TAM subset characterized by an M2-skewed profile and impaired phagocytic activity against tumor cells [197]. Accordingly, the genetic ablation of PD-L1 increases PD-1^+^ TAM phagocytosis in vivo, inhibits tumor growth, and improves the survival of CRC-bearing mice in a macrophage-dependent manner [197]. These observations support PD-1 as a key determinant for limiting anti-tumor immunity and highlight the therapeutic potential of enhancing TAM effector activities in CRC immunotherapy.

To date, the CD47/signal-regulatory protein α (Sirpα) is the best characterized innate immune checkpoint, which regulates phagocytic and cytotoxic activities of myeloid cells. Sirpα is an inhibitory receptor expressed by myeloid cells, which binds CD47, a “don’t eat me” signal physiologically expressed by normal tissue and hematopoietic cells. Sirpα/CD47 axis blocks phagocytosis, preventing the destruction of self-tissues; however, in the tumor context, the upregulation of CD47 by neoplastic cells represents a mechanism to escape immune clearance [198]. Accordingly, in a wide range of human cancers, CD47 expression levels are associated with a worse outcome, and multiple clinical trials have started to evaluate its neutralization by monoclonal antibodies, in particular by combinatory strategies [199,200]. Although in gastrointestinal malignancies, anti-CD47 therapies are still in the early stages of development, the prognostic and therapeutic values of Sirpα/CD47 are being confirmed by a growing number of studies [201]. In CRC patients, single-nucleotide polymorphisms in CD47 [202] and high CD47 expression [203] are found to be associated with poor prognosis and distant metastasis. Interestingly, CD47 expression correlates with both CD44 expression and EMT, suggesting CD47 as a promoter of cancer cell stemness, tumor spreading, and resistance to PD-1/PDL-1 inhibitors [203]. In different CRC models, the expression of the inhibitory immune checkpoint receptor Sirpα in TAM increases during tumor progression, in association with impaired phagocytosis of tumor cells [204]. Mechanistically, CRC-derived lactate induces the expression of Sirpα through the sequential activation of the transcription factors—Ap-2α and Elk-1 [204]. This evidence fosters additional studies to evaluate the therapeutic potential of this phagocytic checkpoint in CRC. In particular, given that synergistic action of Sirpα/CD47 blockade and ICBs has been reported in other tumor types [200], neutralization of Sirpα represents an attractive approach to increase the responsiveness of CRC patients to ICB.

Recently ST2, the only known receptor of IL-33, has emerged as an attractive immune checkpoint for new combinatory strategies. In CRC patients, ST2 has been overexpressed in TAM and associated with low CD8^+^ T cell cytotoxicity and poor outcome [205]. In the preclinical models of CRC, ST2-expressing TAMs increase during tumor progression, promoting the generation of an immunosuppressive TME favorable for CRC growth [205]. Indeed, pharmacological inhibition of ST2^+^ TAMs recruitment by an IL-33 traps fusion protein, or lack of host ST2 significantly reduces CRC growth in a macrophage-dependent manner. Genetic depletion of ST2 also increases the frequency and functions of intratumor CD8+ T cells and acts synergistically with anti-PD-1 checkpoint blockade. Therefore, ST2 is an additional immune checkpoint whose neutralization might be exploited to alleviate the immunosuppressive TME and broaden the number of CRC patients who can benefit from ICB [205].

The diversity and composition of the gut microbiota are found to be key determinants in response to treatment with ICB [206,207,208]. In different epithelial cancers, an abnormal gut microbiome due to the use of antibiotics is associated with ICB resistance [206]. Different studies have demonstrated that FMT of ICB responding patients in germ-free mice enhances the efficacy of anti- PD-L1 therapy, leading to augmented T-cell responses and improved tumor control [207,208]. Metagenomics of patient stool samples at diagnosis have identified a significant association between clinical responses to ICB and the relative abundance of selective bacterial species (e.g., *Bifidobacterium longum, Collinsella aerofaciens, Enterococcus faecium, Akkermansia muciniphila, Bacteroides fragilis, Bacteroides cepacia,* and *Bacteroides thetaiotaomicron*) [206,208]. Interestingly, oral supplementation in non-responder mice, with some of these enriched bacterial species, such as *Akkermansia muciniphila* and *Bacteroides fragilis,* respectively, restores the efficacy of PD-1 and CTLA-4 blockade [206,209]. In CRC patients, the influence of the microbiota on CRC responsiveness to ICBs is still largely unexplored; however, some preclinical evidence has pointed out an association between enteric bacteria and the efficacy of immunotherapeutic approaches based on CpG-oligodeoxynucleotide (CpG-ODN). In subcutaneous CRC models, intra-tumoral injection of CpG-ODN along with neutralization of IL-10 have shown impressive results in conventional mice, but are largely ineffective in microbiota-depleted mice [171]. CpG-ODN triggers TAM to release a copious amount of TNF that leads to a rapid tumor hemorrhagic necrosis [171,210]. Of note, mice showing elevated TNF in response to CpG-ODN show a different composition of the enteric microbiota. Specifically, the abundance of Gram- *Alistipes* and Gram+ *Ruminococcus* bacteria positively correlates with TNF production and response to CpG-ODN, whereas the presence of commensal *Lactobacillus* spp is associated with resistance [171,211].

## 8. Conclusions and Future Perspectives

Independently on CRC origin, TAMs emerge at the crossroads of the inflammatory pathways, driving tumor development and response to therapy. Due to their inherent functional plasticity, macrophage can play a dual role, contributing to anti-tumor immunity or supporting the development of an immunosuppressive TME that promotes tumor progression and resistance to therapy. Deciphering the heterogeneity of TAMs and its relevance in the interplay with both cancer cells and the other cell components of the TME is the challenge to better define the prognostic and predictive value of TAMs, as well as the therapeutic potential of TAM-targeting approaches. New technological advances, including scRNA-Seq, are likely to achieve this and to generate new specific macrophage-centered strategies.

Although chemotherapy remains the first-line treatment of metastatic CRC, therapies targeting TME-modulating factors, such as vascular endothelial growth factor, epidermal growth factor receptor, and immune checkpoint inhibitors, have been shown to be effective in patients with specific subtypes of CRC [212]. In an effort to increase the number of patients who can benefit from ICBs, there is growing interest in developing combination strategies that include conventional therapies, multiple ICBs, or co-stimulatory agonists. In particular, approaches targeting innate and adaptive immune responses promise to generate more potent anti-cancer responses. Within this scenario, neutralization of phagocytosis checkpoints, such as the Sirpα/CD47 axis, can enhance TAM effector activities and might act synergistically in combination with ICB [200]. While anti-CD47 therapies have already shown impressive results in hematologic malignancies, increasing evidence is confirming the prognostic and therapeutic value of the Sirpα/CD47 axis in gastrointestinal cancers [201].

The impact of the microbiota on the outcome of cancer treatment and anti-tumor immunity [46] arises new questions in the CRC field. Given that a specific microbial signature can influence the prognosis of CRC patients and the host’s sensitivity to conventional and ICB-based immunotherapy, manipulation of gut microbiota is a potential strategy for CRC treatment. FMT is an approach to restore gut microbial homeostasis, which has been found effective for the treatment of resistant *Clostridium difficile* infection [213], thus opening promises for other gastrointestinal disorders, such as IBD and CRC.

Further, probiotics can exert beneficial immunomodulatory and anti-cancer activities, and therefore, they could be exploited for both CRC prevention as well as to improve clinical response and reduce the collateral effects of anti-tumor treatments [214]. Notably, several probiotics modulate macrophage functions, either limiting the production of inflammatory mediators (e.g., *Lactobacillus fermentum*) or enhancing macrophage activation (cell surface molecules of *Lactobacillus* strains, bacterial extracts of *Lactobacillus rhamnosus GG* and *Bifidobacterium adolescentis*) [215,216]. The former could be useful in the CRC prevention setting, whereas the latter might be exploited in combination with immunotherapy. Accordingly, preclinical studies have demonstrated that oral administration of *Alistipes shaii*, in antibiotics pre-treated mice, reestablishes the production of TNF by TAMs during anti-IL-10/CpG-ODN therapy [171]. Of note, in a preclinical model of CAC, the combined administration of *Lactobacillus acidophilus* lysates with anti-CTLA-4 enhances anti-tumor immune responses, leading to a significant reduction of CRC development. In comparison to ICB alone, the administration of the probiotic reshapes tumor immune infiltrate by increasing tumor-infiltrating CD8 +T cells and effector memory T cells and by reducing immunosuppressive T regulatory cells and M2 macrophages [217].

Finally, the tumor homing capacity of some bacteria can be exploited to enhance anti-tumor immunity. For example, in an orthotopic tumor model of CRC, it has been demonstrated that engineered attenuated *Salmonella* strains expressing TLR5 ligand activate an immune response, leading to a significant anti-tumor activity [184]. The direct modulation of immune cells through the administration of bacterial products is, therefore, an alternative strategy to engage anti-cancer immunity. Ligands for TLRs or other innate receptors are being developed for clinical use in combination with cancer therapies [218].

In addition to bacteria, the human microbiota contains a virome and the mycobiome. Although our understanding of both is in its infancy, growing evidence indicates that alterations in the enteric virome and mycobiome are associated with CRC, so a better understanding of the composition of microbial communities may open new strategies for therapeutic modulation of the microbiota.

## Figures and Tables

**Table 1:** Markers of mouse and human macrophages polarized activation are depicted (*CCL18 is a human-specific marker).

	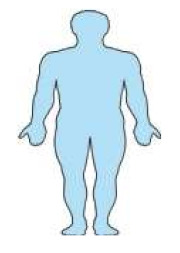 Human	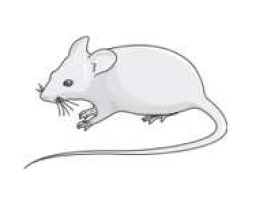 Mouse
Pan-macrophage	CD68^+^, CD14^+^, CD16^+^, HLA-DR^+^ CD64^+^	F4/80^+^, CD68^+^, CD64^+^ CXC_3_R1^int/hi^, MHCII^+^, CD11b^+^, Ly6C^low^
M1-like (anti-tumoral)	CD40, CD80, CD86, NOS2 TNFα, IL1β, IL6, IL12, IL23 CXCL9, CXCL10, CXCL11	
M2-like (pro-tumoral)	CD163, CD204, CD206, CD209, CD301, Dectin-1, IDO, ARG1, HO-1 IL10, TGFβ, CCL17, CCL18*, CCL22	

## Abbreviations

4-HNE	4-hydroxy-2-nonenal
5-FU	5-fluorouracil
Arg1	Arginase 1
CAC	Colitis-associated cancer
CCL	Chemokine (C-C motif) ligand
CCR	Chemokine (C-C motif) receptor
CD	Cluster of Differentiation
COX-2	Cyclooxygenase-2
Cpg-ODN	CpG-oligodeoxynucleotide
CRC	Colorectal cancer
CSF1R	Colony-stimulating factor 1 receptor
CTLA- A	Cytotoxic T-lymphocyte-associated protein 4
CXCL	Chemokine (C-X-C motif) ligand
DCLK1	Doublecortin-like kinase 1
DCs	Dendritic cells
dMMR	DNA mismatch-repair
DSS	Dextran sulfate sodium
EMT	Epithelial-mesenchymal transition
EP	Prostaglandin E receptor
FadA	Fusubacterium adhesin A
Fap	Fatty-acid-binding protein 2
FMT	Faecal microbiota transplantation
HLA	Human leukocyte antigen
HLA-DR	Major histocompatibility complex, class II, DR
IBD	Inflammatory bowel disease
ICBs	Immune checkpoint blockers
ICD	Immunogenic cell death
IDO	Indoleamina 2,3-diossigenasi
IFN	Interferon
IKK	Nuclear factor NF-kappa-B inhibitor kinase
IL	Interleukin
IL1R	Interleukin 1 receptor
LPS	Lipopolysaccharide
MDSCs	Myeloid-derived suppressor cells
M-MDSCs	Monocytic myeloid-derived suppressor cells
MSI-H	Microsatellite instability-high
MSI-L	Microsatellite instability-low
NF-κB	Nuclear factor kappa-light-chain-enhancer of activated B cells
NK	Natural killer cells
NO	Nitric oxide
NOS2	Nitric oxide synthase 2
NOX 2	NADPH oxidase 2
NSAIDs	Nonsteroidal anti-inflammatory drugs
OMVs	Outer membrane vesicles
PD-1	Programmed-death protein 1
PD-L1	PD-1 ligand
PGE2	Prostaglandin E2
POL	DNA polymerase
Rad DRNS	Arginine-inhibitable adhesinReactive nitrogen species
ROSSCFA	Reactive oxygen speciesShort-chain fatty acids
scRNA-Seq	Single cell RNA-Seq
Sirpα	Signal-regulatory protein α
Stat3	Transducer and activator of transcription 3
TAMs	Tumor-associated macrophages
TGF	Transforming growth factor
Th	T helper
TLR	Toll-like receptor
TMB	Tumor mutational burden
TME	Tumor microenvironment
TNF	Tumor necrosis factor
TRMs	Tissue-resident macrophages
